# Handling Big Data Scalability in Biological Domain Using Parallel and Distributed Processing: A Case of Three Biological Semantic Similarity Measures

**DOI:** 10.1155/2019/6750296

**Published:** 2019-01-27

**Authors:** Ameera M. Almasoud, Hend S. Al-Khalifa, Abdulmalik S. Al-Salman

**Affiliations:** College of Computer and Information Sciences, King Saud University, Riyadh, Saudi Arabia

## Abstract

In the field of biology, researchers need to compare genes or gene products using semantic similarity measures (SSM). Continuous data growth and diversity in data characteristics comprise what is called big data; current biological SSMs cannot handle big data. Therefore, these measures need the ability to control the size of big data. We used parallel and distributed processing by splitting data into multiple partitions and applied SSM measures to each partition; this approach helped manage big data scalability and computational problems. Our solution involves three steps: split gene ontology (GO), data clustering, and semantic similarity calculation. To test this method, split GO and data clustering algorithms were defined and assessed for performance in the first two steps. Three of the best SSMs in biology [Resnik, Shortest Semantic Differentiation Distance (SSDD), and SORA] are enhanced by introducing threaded parallel processing, which is used in the third step. Our results demonstrate that introducing threads in SSMs reduced the time of calculating semantic similarity between gene pairs and improved performance of the three SSMs. Average time was reduced by 24.51% for Resnik, 22.93%, for SSDD, and 33.68% for SORA. Total time was reduced by 8.88% for Resnik, 23.14% for SSDD, and 39.27% for SORA. Using these threaded measures in the distributed system, combined with using split GO and data clustering algorithms to split input data based on their similarity, reduced the average time more than did the approach of equally dividing input data. Time reduction increased with increasing number of splits. Time reduction percentage was 24.1%, 39.2%, and 66.6% for Threaded SSDD; 33.0%, 78.2%, and 93.1% for Threaded SORA in the case of 2, 3, and 4 slaves, respectively; and 92.04% for Threaded Resnik in the case of four slaves.

## 1. Introduction

Massive data is generated daily from multiple sources such as electronic devices or the Internet; network sensors and healthcare and laboratory equipment; and sources of mobile data. Data generated from the Internet comes from social networking sites, governments, or large companies such as Google and Yahoo. In recent years, these data sources have grown continuously; traditional approaches to data management cannot handle this growth. This phenomenon is called “big data.”

Laney [1] defined challenges present in big data management in three dimensions (a.k.a., the 3Vs): volume, variety, and velocity. Volume refers to the increasing size of data. Variety refers to the types of data including text, graphs, images, video, audio, and other types. Velocity means that data are generated continuously as a stream at high speeds and needs to be processed as they are generated. Fan et al. [2] added two more Vs to this model: variability and value. Variability means there are changes in data structure and interpretation. Value is the business value that gives a competitive advantage to the organization. Volume and velocity were the focus of previous research; the variety of available data worldwide has received less attention. Abawajy [3] discussed dimensions in the variety of big data, terming them structure diversity, content diversity, source diversity, and processing diversity. Structure diversity includes three types of data: structured data, semistructured data, and unstructured data. Content diversity means data are single-media data, multimedia data, or graph data. Source diversity means data are machine-generated, human-generated, or process-generated. Finally, processing diversity represents the data processing types, namely, batch processing, stream processing, interactive processing, or graph processing.

Genetics is one of the biggest sources of big data. A single sequence of human genome is approximately 140 gigabytes; therefore, storing and comparing human genomes require more than a personal computer and online file-sharing applications. The European Bioinformatics Institute (EBI), one of the most important repositories of big data in biology, stores more than 20 petabytes of data on genes, proteins, and small molecules; one petabyte is 10^15^ bytes. Genomic data represents two petabytes of that, and this number is doubled every year. Biology labs access approximately one terabyte (10^12^ byte) of big data stored at EBI or the National Center for Biotechnology Information daily and generate more new data. Therefore, small labs are also generators of big data [4].

Biological data increase not only in size but also in diversity [5]. Biological data are produced via a wide range of procedures; each procedure generates various pieces of information such as those on genetic or protein interactions. These data are analyzed within or across different heterogeneous sources, providing information that cannot be found from analyzing the literature or individual data sources. Therefore, it is important that companies and researchers have the ability to mine and analyze big data to find information, establish patterns, and form hypotheses.

Calculating semantic similarity is essential for comparing genes and gene products. A semantic similarity measure is a function that takes two GO terms or two sets of terms representing the annotations of two entities and returns a numerical value representing the closeness in meaning between them [6]. Standard SSMs such as Palmer's [7], cosine similarities [7, 8], and semantic proximity [9, 10] are suitable for some fields of study but are inaccurate for calculating semantic similarity between objects in other fields. In the field of biology, for example, comparing GO annotation terms is not enough; therefore, semantic similarity is measured by comparing features that describe the objects and the hierarchal relationships between these features [11–13]. Consequently, some SSMs are defined specifically for biology to measure the similarity between genes and gene products. A biological SS measure can be used to compute: similarity between gene ontology (GO) terms (term similarity), similarity between GO products (where each product is annotated with a set of GO terms), and gene product similarity.

There is no standard approach to determine the best similarity measures for each application; therefore, literature and recent surveys [14–16] compare and test SSMs. Recent reviews indicate that Resnik is the best SS measure in certain settings, followed by SSDD and SORA. To the best of our knowledge, no previous studies applied these similarity measures to big data. However, using semantic similarity measures to analyze large sets of biomedical data is addressed in [17], in which they used parallel computation on a multicore processor, and in [18], where GO information was stored in a hash table to avoid repeatedly traversing the GO graph, thereby improving computational efficiency. Here, we aimed to enhance the three best SSMs designed for biology (Resnik, SSDD, and SORA), enabling them to handle big data volume using a distributed processing system. Biological SSMs cannot handle big data. Therefore, a distributed processing system can be used to split data into multiple partitions. SS measures are then applied to each partition. This manages big data scalability and avoids computational problems, leading to good performance. Consequently, in this study, we investigated how using a distributed processing system can improve the performance of Resnik, SSDD, and SORA in the field of biology.

The rest of this paper is organized as follows.  Section 2 introduces a background about gene ontology (GO) and Semantic Similarity Measures (SSMs).  Section 3 describes in detail the materials and methods for enhancing the best three biological semantic similarity measures.  Section 4 discusses and analyzes the results.  Section 5 provides the conclusions and future directions.

## 2. Background

### 2.1. Gene Ontology

**Figure 1:**
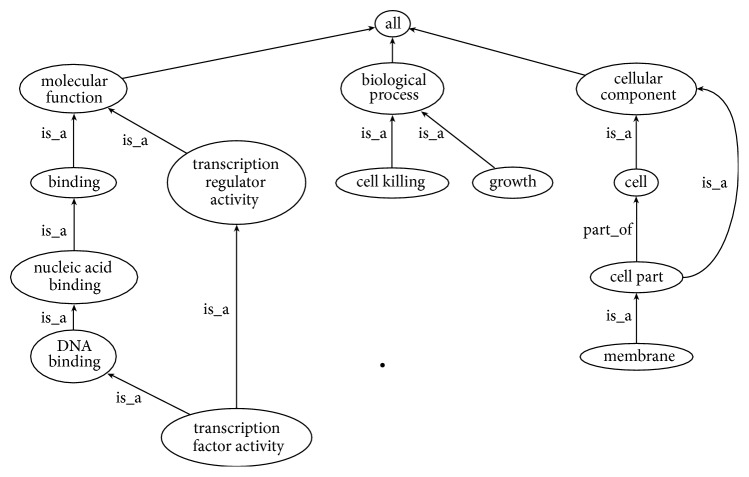
Section of GO graph showing biological process (BP), molecular function (MF), and cellular components (CC) and some of their descendants [16].

GO is a valuable resource in bioinformatics. GO provides a structured, precisely defined, and controlled vocabulary to describe genes and gene products according to three categories: biological process (BP), molecular function (MF), and cellular components (CC) [19]. Each of these categories is represented by a separate ontology of terms such as rooted Directed Acyclic Graph (rDAG) [20] (Figure 1). Each term in GO is associated with annotations describing MF, biological role, and localization. Annotation can be computationally inferred, such as Inferred from Electronic Annotation (IEA), or experimentally determined, which is indicated by an Evidence Code (EC). EC is more reliable than IEA in representing the type of process that generates the annotation [15].

### 2.2. SSM

A semantic similarity measure (SS measure) is a function that takes two GO terms, or two sets of terms representing annotations of two entities, and returns a numerical value representing the closeness in meaning between them [6]. An SS measure can be used to compute similarity between GO terms (term similarity), similarity between GO products (where each product is annotated with a set of GO terms), and gene product similarity. Term similarity and gene product similarity are described below [21].

#### 2.2.1. Term Similarity

This SS measure was developed by Rada et al. [22], who proposed a metric called distance to measure the distance between two concepts in a graph via the shortest path between these concepts. Distance has some limitations. It considers that all edges in the graph have the same weight. This is not the case in GO, where edges may have different weights even if they are at the same level. Moreover, it takes the shortest path between two nodes regardless of their distance to the root (depth). Previous studies used two methods to solve these issues. The internal method was to calculate the semantic similarity between two concepts based on GO structure. The external method was to calculate semantic similarity based on external corpora.

- Internal methods resolved the aforementioned issues by considering the depth of the lowest common ancestor (LCA) between terms [23], distance to the nearest leave node [24], depth of the distinguished GO subgraph [25], and distance to the LCA between terms with a number of subclasses [26].
- External methods were developed by Resnik [27], where the semantic similarity between two terms is calculated based on Information Content (IC) and GO taxonomy structure. IC measures the similarity between two concepts by measuring how much information they share. The IC of a concept is acquired by calculating the probability of the occurrence of the concept in a selected corpus. As described in [28], uniformly scaling the IC values simplifies interpretation. There are two methods for applying IC to the common ancestor of two concepts: considering the most informative common ancestor (MICA) with the highest IC [27] or considering all the disjoint common ancestors (DCA) [29–31]. Therefore, the similarity between two concepts can be the IC of MICA [32] or the combined IC of MICA and that of the two concepts, which are weighed according to the IC value of MICA [33].
- Hybrid methods combine both internal and external methods, such as combining the IC-based strategy with the edge [34], number of descendants [35], depth and descendants [36], or entropy [37].

#### 2.2.2. Gene Product Similarity

A gene product can be annotated by several GO terms. To calculate SS measure for these terms, pairwise or groupwise methods can be used:

- Pairwise method calculates individual semantic similarity among all terms annotating two gene products and then calculates the average, maximum, minimum, or sum for all the pairs of terms or only for the best-matched pair of each term. For example, average (AVG) [32] calculates the average of all pairwise similarities; maximum (MAX) [38] calculates the maximum of all pairwise similarities; best match average (BMA) [39] calculates the average of the best-matched pairs; and FunSim [33] combines two semantic similarities by finding AVG, MAX, or BMA values and combining them in a nonlinear approach. IC-based semantic similarity [40] creates averages for the best-matched pairs. FuSSiMeG [41] is similar to MAX, but it weighs the IC pairwise similarities of the terms, after which the term with maximum IC weight is selected.
- Groupwise methods calculate semantic similarity using a set, graph, or vector approach:
  - In the set method, groupwise methods encompass set-based techniques with respect to all direct annotations. The main disadvantage of this method is that it does not take into account the shared ancestry between GO terms.
  - In the graph method, the direct and indirect annotations of gene products are represented as a graph, and set-based or graph-matching methods are used afterward to calculate semantic similarity. This method is better than the set method because it considers all direct and indirect annotations.
  - With vector methods, gene products are represented in a vector space, where each term is represented as a dimension; similarity is calculated using the vector similarity measure.

Several previous studies combined groupwise approach with the IC of terms. One study considered using the IC of terms to perform similarity computations, such as in simGIC [28], which compares two sets based on an IC-weighed Jacquard similarity. Additionally, IC can be used as a scalar value, such as in InteliGO [42], which combines the IC value and evidence content of annotations. Moreover, IC can be used to compute the IC value of shared subgraphs [27].

#### 2.2.3. The Best SS Measure

There is no standard approach to determine the best similarity measure for each application; therefore, literature and recent surveys [14–16] have compared and tested SSMs. These reviews indicate that Resnik is the best SS measure in certain settings, followed by SSDD and SORA. Resnik [27] determines the semantic similarity of a protein based on the IC of the MICA. Additionally, the Best-match-avg function (Resnik) [27] determines the semantic similarity of proteins based on the average of best-matched terms. Shortest semantic differentiation distance (SSDD) [26] measures the semantic similarity of GO terms based on the “totipotency” concept, where each term is assigned a value representing its distance to the root and the number of descendants at each level in that path. The similarity between two terms is the smallest sum of “totipotency” along the path between them. In SORA [36], the IC value of the term and those of its inherited and extended terms are calculated separately and then combined with one IC value using term-set similarity. The similarity between two genes is the average of the IC values of their term sets.

## 3. Materials and Methods

In our study, enhancing Resnik [27], SSDD [26], and SORA [36] to be able to handle big data volume is based on distributed processing. In distributed processing, SSMs are used with a master-slave architecture, such that one device has unidirectional control over other devices. Our proposed process consists of three steps: the first two steps are the responsibility of the master node, and the third step is the responsibility of the slaves.

1. Split GO: this step is used as the initial step to divide GO into N splits, ensuring to render the similarity within each split very high, reduce the percentage of shared descendants with other splits, and make the split as balanced as possible.
2. Data clustering: this step is used to cluster or split data input into N splits based on the N splits generated during the first step. The resulting clusters are then sent to one of the slaves.
3. Semantic similarity calculation: in this step, Resnik, SSDD, or SORA is applied to the input data cluster; the results are then sent back to the master node.

There are two methods for using these enhanced SSMs with the distributed system. The first method is to divide the input data equally among the number of slaves. The second method is to divide the input data based on their similarity using split GO and data clustering algorithms. These two methods were applied to compare the average and total time used by enhanced Resnik, SSDD, and SORA. The details of each step are discussed in the following subsections.

### 3.1. Split GO

**Figure 2:**
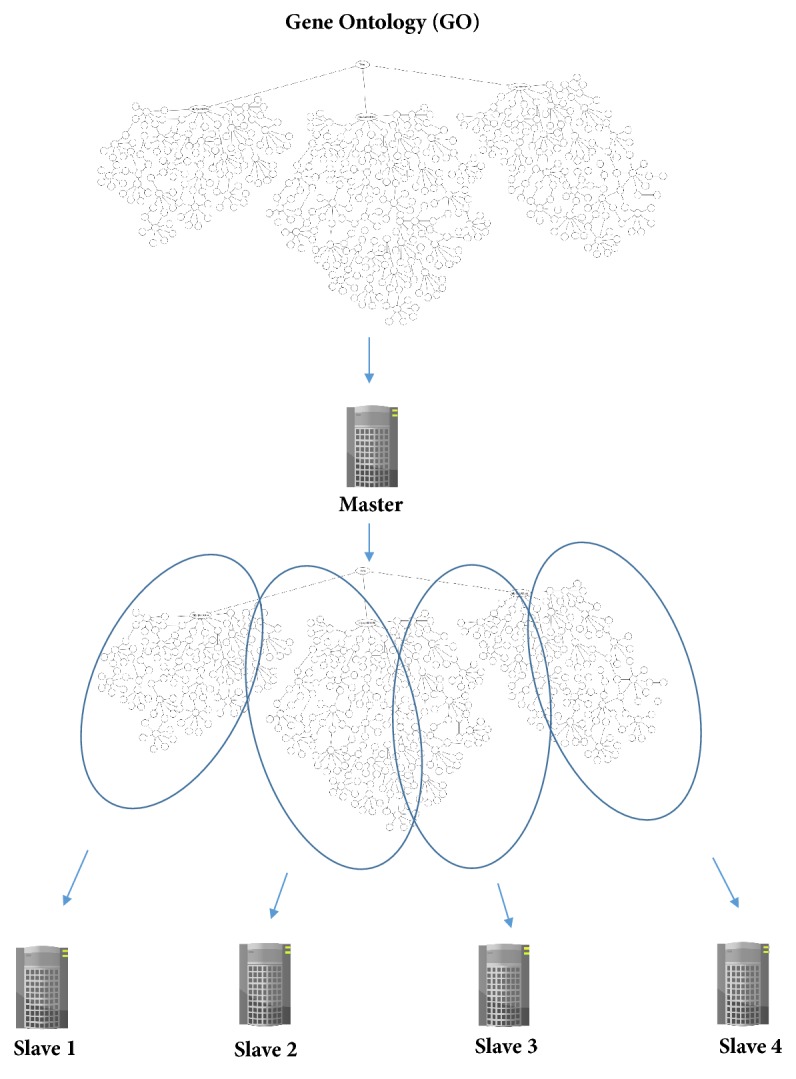
Example of splitting gene ontology (GO) into four splits.

We tested several methods for splitting GO into several parts to be used in the distributed system. In this approach, each split is assigned to one of the slave systems. The main goal of our approach was to divide GO into N splits, ensuring a very high similarity within each split, reducing the percentage of shared descendants with other splits, and rendering the split as balanced as possible. Using these methods, the input is GO and the number of splits is N. The master node is responsible for dividing GO into N splits (one split for each slave node).  Figure 2 illustrates the division of GO into 4 splits and assignment of each split to one slave.

**Figure 3:**
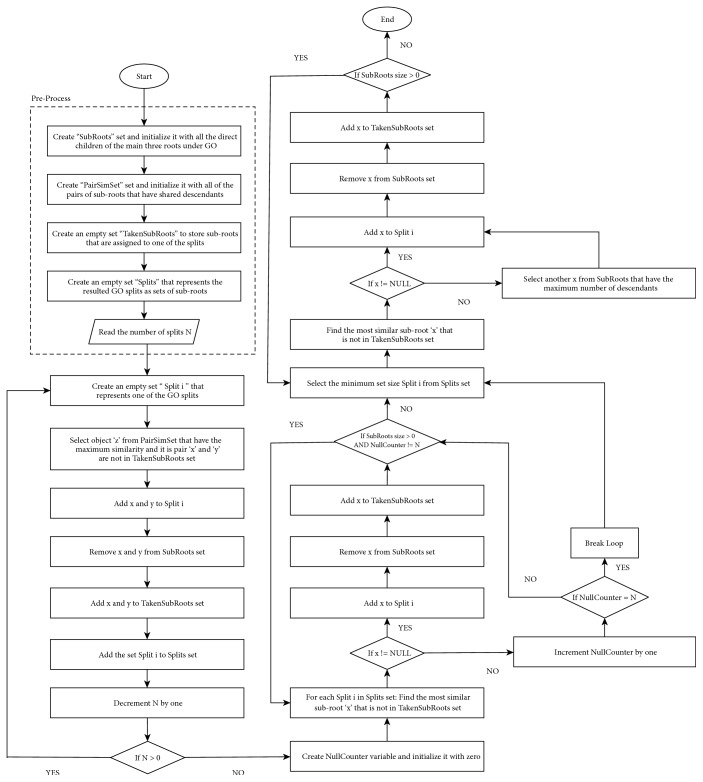
Flowchart of GO splitting algorithm.

The proposed methods are as follows: (1) split graph by the main three roots (molecular function, cellular component, and biological process); (2) split graph by roots or subroots (there are a total of 65 subroots, with 25 subroots under root1, 21 subroots under root2, and 19 subroots under root3); or (3) split graph by subroots only. These methods are first used to initialize each split with one of the largest root/subroot, continuing until no roots/subroots remain. To avoid the issue of balance in our proposed methods, we initialized each split with a pair of the most similar subroots, and continued adding the most similar subroots to each split until no more similar subroots remained. Then, we selected the smallest split, found the most similar subroot from the remaining subroots, and assigned it to this split. If there were no more similar subroots for this split, we added one of the largest remaining subroots and repeated this process until there were no remaining subroots. This method increases the similarity of subroots within each split and reduces the overlap between the splits, which is our goal. This method can be used as an initial step before partitioning the input data (pairs of genes) on the distributed systems in order to calculate the similarity among gene pairs. A flowchart of the algorithm used in this method is shown in Figure 3.

### 3.2. Data Clustering

**Figure 4:**
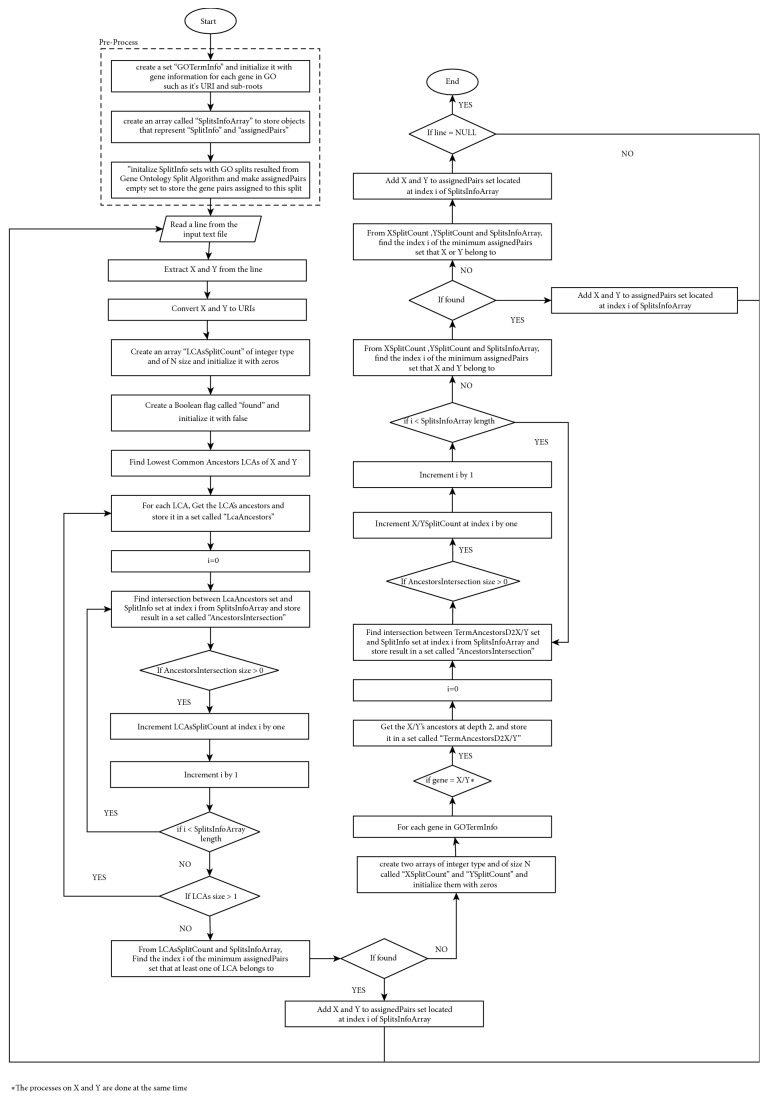
Flowchart of the data clustering algorithm.

The data clustering step is used for clustering data input into N splits. The process starts at the master node. First, we took the N splits generated from the gene ontology splitting algorithm and a text file of gene pairs. Then, we clustered the input file into N clusters, based on the data clustering algorithm, before sending each cluster to one of the slaves. In this algorithm, a gene pair (X, Y) is added to the minimum cluster, ensuring that at least one of its LCAs belongs to its split. If the LCA does not belong to any split, then the algorithm adds the pair to the minimum cluster containing the gene X and Y. If the genes X and Y belong to different splits, the algorithm adds the pair to the minimum cluster that contains X or Y. LCA is used to group the neighbors/most similar gene pairs to speed the similarity calculation at the end. LCA plays the main role in the algorithm because it is used in the similarity calculation employed by many SSMs such as Resnik and SSDD. On the other hand, SORA does not use LCA directly but it depends on the IC value of the term and those of its inherited and extended terms (neighbors/most similar genes). Also, LCA represents the nearest ancestors to both X and Y. If LCA is near the root, the difference between X and Y is very high; however, if LCA is farther from the root, the difference between X and Y is low [26]. A flowchart of the data clustering algorithm is shown in Figure 4.

### 3.3. Semantic Similarity Calculation

In this step, each slave applies one of the SSMs (Resnik, SSDD, or SORA) to its assigned data cluster. So, each slave calculates the similarity of each gene pair and then sends the results to the master node. Then, the master node combines the results into one file. To enhance the performance of these SSMs, we suggest using threads, as detailed in the following subsections.

### 3.4. Enhanced Semantic Similarity Measure in the Distributed System

Our proposed framework is composed of one master and N slaves, which communicate with each other across socket programming. Data are shared among the master and slave nodes via Samba file and print services [43] located at the master node. When a slave starts running, it can load GO from the Samba server, open a socket, and wait for any request from the master node. When all slaves are running and ready, the master node reads the input data from the Samba server, divides them into N splits, sends input data splits to slaves (one split for each slave), and waits for the response. When responses are sent to the Samba server at the master node, the results can be combined into one output file. The master node splits input data by dividing the total input equally into N splits, allocated to N blocks, which is the number of slaves. If there are less than N remaining lines not assigned to any block, the user can add them to the last block. Finally, to each slave, the master node sends the path to the original input file, the number of lines in the block, and the offset of the first line in the block. The master node can also divide the data based on their similarity by using the GO Splitting algorithm to divide GO into N splits, then clustering the input data via data clustering algorithm according to these splits. Then, each data split is placed into a separate file before the file paths are sent to slaves.

We tested the original Resnik, SSDD, and SORA SSM; then, we used parallel processing to enhance the performance of these SSMs. In Resnik, threads are introduced at the points of finding the ancestors of gene pairs X and Y. In SSDD, threads are introduced at the points of finding the T values for each vertex in the path from X/Y to LCA. In SORA, threads are introduced at the points of calculating the IC value of X descendants, Y descendants, and the union of X and Y descendants. The performance of the threaded measures is shown in the results section.

### 3.5. Implementation and Testing

We validated the performance of the GO split algorithm, data clustering algorithm, enhanced SSMs (Resnik, SSDD, and SORA), and applied these enhanced methods in the distributed system. Implementation and testing were conducted using the following settings and equipment:

- Equipment:
  - Dell PowerEdge T620 server with a VMware Workstation Pro 14 software to create a set of five virtual machines (VM); each machine runs on Ubuntu 16.04 LTS, Intel® Xeon® processor E5-2600 product family × 4 processors, and 8 GB of memory. One VM works as a master and the rest work as slaves.
- Programming language:
  - JAVA programming language version 1.8.
  - Libraries:
    1. Semantic measure library and toolkit (SML) [17] to read and process the GO.
    2. JCIFS library [44] to access and manage shared data on a Samba Server installed on the master node using JAVA.
- Input:
  - GO [45] as input in Open Biomedical Ontologies (OBO) file format [46]; it is composed of 36638 genes.
  - Gene pairs are written in a text file, where genes are generated randomly to create six samples with different sizes. Sizes range from 10 to 1000000 (increased by a factor of 10).
    1. Due to the diversity in the number of descendants under the main three roots in the original GO and to ensure that the generated samples are distributed equally among the GO genes, we generated 0.08% of the pairs from the descendants under the smallest root, 0.26% of the pairs from the descendants under the medium root, and the rest of the pairs from the descendants under the largest root for each sample. These percentages relate the number of descendants under each root to the total number of descendants under GO.
- SSM:
  - The original SSM (Resnik, SSDD, and SORA).
  - Enhanced versions of the SSM (Threaded Resnik, Threaded SSDD, and Threaded SORA).
- Algorithms:
  - GO split algorithm to generate N GO splits, where N ranged from 1 to 4, because in our settings we can have 2, 3, or 4 slaves.
  - Data clustering algorithm to divide input data into N clusters based on the results of the GO split algorithm.
- Test cases:
  - Case 1: testing the performance of the enhanced SSMs. This test is performed on a single virtual machine to measure the following:
    1. Performance of original SSMs (Resnik, SSDD, and SORA).
    2. Performance of enhanced SSMs (Threaded Resnik, Threaded SSDD, and Threaded SORA).
    3. Comparison of the performance of enhanced SSMs with the original SSMs.
  - Case 2: testing the performance of enhanced SSM in the distributed system. This test is conducted three times using one master and two slaves, one master and three slaves, and one master and four slaves. This test is used to measure the following:
    1. Performance of enhanced SSMs (Threaded Resnik, Threaded SSDD, and Threaded SORA) if the input data are divided equally.
    2. Performance of enhanced SSMs (Threaded Resnik, Threaded SSDD, and Threaded SORA) if the input data are divided by their similarity using the GO split and data clustering algorithms.
    3. Comparison of the performance of enhanced SSMs (Threaded Resnik, Threaded SORA, and Threaded SSDD) when the data are divided equally, when data are divided based on their similarity.

In all these cases, performance is the total and the average time required to calculate the semantic similarity of the gene pairs. In our opinion and based on the experiment in [47], the average time is more important than the total time because average time reflects the time required to measure semantic similarity for the majority of gene pairs. That is not the case with total time, which can increase with values that are far from the average value, when calculating semantic similarity of certain genes. In assessment 1, Improvement Percentage (IP) of average/total time was measured according to

(1) IP=Threaded  SSM  Average/Total  Time  nsOriginal  SSM  Average/Total  Time  ns∗100−100

**Figure 5:**
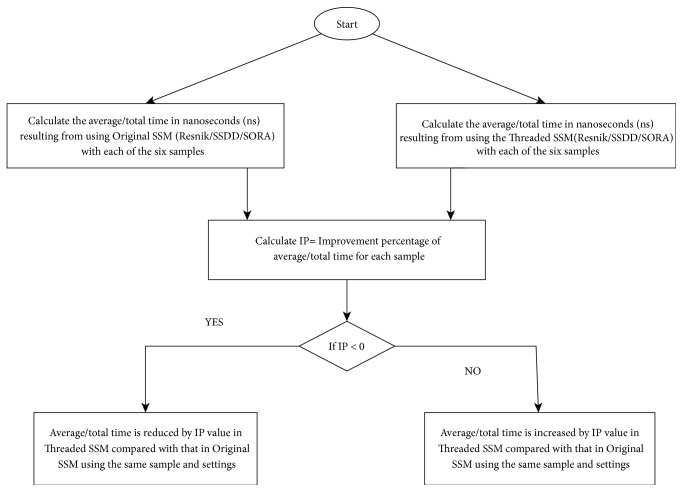
A flowchart of testing the performance of enhanced SSMs.

The Improvement Percentage value of negative x indicates that an average/total time in nanoseconds (ns) was obtained using Threaded SSM. The time was reduced by this x value compared with the average/total time required by the original SSM using the same sample and settings. The Improvement Percentage value of positive x indicates that average/total time in ns was obtained using Threaded SSM. The time was increased by this x value compared with average/total time required by the original SSM using the same sample and settings. The average of the IP is then measured to find the mean value of the IPs.  Figure 5 shows a flowchart of this procedure.

In assessment 2, IP of average/total time is measured according to

(2) $$\begin{array}{c} IP=\left(\;\frac{Threaded\;\;SSM\;\;Average/Total\;\;Time\;\;\left(ns\right)\;\;where\;\;input\;\;divided\;\;by\;\;their\;\;similarity}{Threaded\;\;SSM\;\;Average/Total\;\;Time\;\;\left(ns\right)\;\;where\;\;input\;\;divided\;\;equally}*100\;\right)-100 \end{array}$$

**Figure 6:**
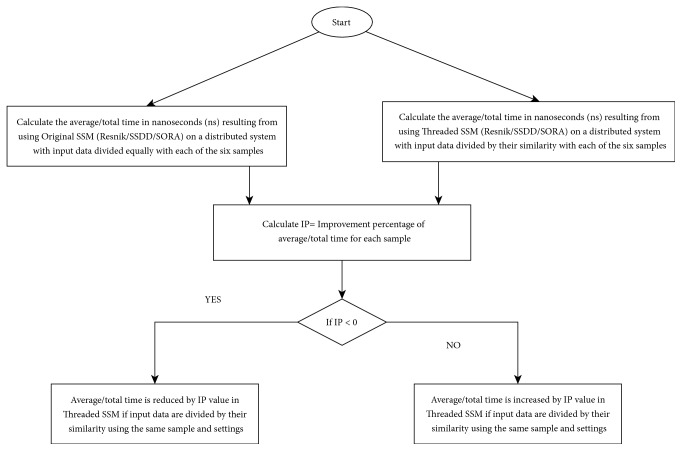
A flowchart of assessing the performance of enhanced SSMs in the distributed system.

If the Improvement Percentage value is negative x, that means average/total time in nanosecond (ns) was obtained using Threaded SSM with input data divided by their similarity via GO split and data clustering algorithms were reduced by x value. If the Improvement Percentage value is positive x, that means that average/total time was increased by the x value. The increases and decreases in average and total time were compared with average/total time obtained using Threaded SSM with input data divided equally and using the same sample and settings. Also, the average of the IP is measured to obtain the mean value of the IPs.  Figure 6 shows a flowchart of this assessment. Detailed results of these assessments are shown in the following sections.

## 4. Results and Discussion

### 4.1. Performance of Enhanced SSMs

- *Threaded Resnik*. Our results show a reduction in the average time required to calculate the Resnik semantic similarity between each pair of genes (Table 1). The average reduction percentage in average time was 24.51 % of that obtained using original Resnik Conversely, the total time required to calculate the semantic similarity measure in Resnik fluctuated; total time decreased in some test samples (such as in sample size=10, 100, 10000) and increased in others (Table 2). The average reduction percentage of the total time was 8.88%.
- *Threaded SSDD*. Introducing threads in SSDD reduced the average and total time. The average reduction percentage of average time was 22.93%, and average reduction percentage of total time was 23.14% (Tables 3 and 4).
- *Threaded SORA*. As in Resnik and SSDD, threads drop the average and total time of calculating semantic similarity.  Table 5 shows that the average reduction percentage of the average time was 33.68%. Also, the average reduction percentage of total time was 39.27% as shown in Table 6. Unlike Resnik and SSDD, SORA requires more memory to measure similarity. For example, with input size of 100000, it took 48 hours to find the similarity of 38045 pairs using original SORA and of 38098 pairs using the threaded version. When input size equals 1 million, it took 61 hours to find the similarity of 139487 pairs using original SORA and of 148182 pairs using the threaded version. In these two cases, the reduction percentage of the total time was approximately 0.14% and 5.87%, respectively.

Introducing threads in Resnik, SSDD, and SORA reduced the time of calculating semantic similarity between gene pairs and improved the performance of these SSMs. The reduction percentage of average time was 24.51% for Resnik, 22.93% for SSDD, and 33.68% for SORA. The reduction percentage of total time was 8.88% for Resnik, 23.14% for SSDD, and 39.27% for SORA.

### 4.2. Performance of Enhanced SSMs in the Distributed System (Input Data Divided Equally)

- *Threaded Resnik*. In the distributed system, applying Threaded Resnik and dividing input data equally dramatically reduced total time. The average reduction percentage increased with increasing the number of slaves. The average total time was reduced by 73.93% in the case of 2 slaves, by 80.65% in the case of 3 slaves, and by 82.29% in the case of 4 slaves (Table 7). The average reduction percentage of total time was reduced because data were distributed, and slaves worked in parallel. However, this does not reduce the average time of calculating the semantic similarity between pairs, unlike the case with total time (Table 8). This is because average time is staggered; it is reduced in some cases and increased in others. Additionally, there is an enormous increase in average time when the number of slaves is increased.
- *Threaded SSDD*. Similar to the results obtained using Threaded Resnik, using Threaded SSDD with a distributed system and input data divided equally reduced the average reduction percentage of total time (Table 9). The reduction was increased by increasing the number of slaves. The average total time was reduced by 59.86%, 65.34%, and 68.19% for 2, 3, and 4 slaves, respectively. Conversely, the average reduction percentage in the average time required for calculating similarity via Enhanced SSDD was markedly increased by increasing the number of slaves (Table 10).
- *Threaded SORA*. The results for Threaded SORA were similar to those obtained with Threaded Resnik and Threaded SSDD in a distributed system with input data were divided equally. The average total time of calculation in Threaded SORA was reduced by 65.01, 72.31, and 66.09 % for 2, 3, and 4 slaves, respectively (Table 11). As we mentioned previously, SORA needs a lot of memory to complete the semantic similarity measure in the Original SORA and Threaded SORA (for input sample sizes of 100,000 and 1,000,000 genes). In the distributed system, with input data divided equally for the same input samples, some slaves finished early; others continued working for a long time until we stopped the test due to suspension. Conversely, the average percentage of total calculation time increased notably (Table 12).

Using the Threaded versions of Resnik, SSDD, and SORA and dividing input data equally dramatically reduced total time. In contrast, the average time of calculating similarity increased markedly by increasing the number of slaves.

### 4.3. Performance of Enhanced SSMs with Input Data Divided by Their Similarity

- *Threaded Resnik*. In the distributed system, with input data divided by their similarity and using our GO split and data clustering algorithms, Threaded Resnik reduced the average time in the case of 4 slaves and sample of size 10,000; average time was increased, however, in the remaining cases, as shown in Table 13. Conversely, total time was decreased with sample size of 10 and 100 and using 2 and 3 slaves, and with sample size of 100 when using 4 slaves. In the remaining cases, total time was increased gradually by increasing the sample size and the number of slaves, as shown in Table 14.
- *Threaded SSDD*. The average time obtained with Threaded SSDD in the distributed system, with input data divided by their similarity, was reduced in the case of 2 slaves and sample size ranging from 10 to 100,000, but increased with sample size of 1,000,000. In the case of 3 slaves, average time increased notably at sample size 10, decreased gradually to a lower value, and then increased again at sample size 1,000,000 (Table 15). In the case of 4 slaves, the average time was markedly increased with sample size of 10. Average time was then reduced gradually until it was lower than the average time obtained with original SSDD, 4 slaves, and sample size of 100000. Average time then increased again with sample size of 1000000. The average reduction percentage of total time was 46.10%, 59.26%, and 48.19% with 2, 3, and 4 slaves, respectively (Table 16).
- *Threaded SORA*. Using Threaded SORA with a distributed system and input data divided by their similarity reduced total time (Table 17). The average reduction percentage of total time was 80.07%, 83.25%, and 84.24% using 2, 3, and 4 slaves, respectively.  Table 18 shows a decrease in average time using 2 slaves and sample size of 100 to 10000. Average time was also decreased using 3 and 4 slaves with a sample size of 10000. However, the average time was considerably increased in the rest of the cases.

Using the Threaded versions of Resnik, SSDD, and SORA with input data divided by their similarity produced different behaviors in each case. In Threaded Resnik, the average time was unexpectedly increased. In most cases, total time was gradually increased by increasing the sample size and number of slaves; however, in a few cases, total time was reduced. In Threaded SSDD and SORA, the average time was decreased in some cases and increased in others. Total time was reduced by 46.10%, 59.26%, and 48.19% using Threaded SSDD and by 80.07%, 83.25%, and 84.24% using Threaded SORA, with 2, 3, and 4 slaves, respectively.

### 4.4. Comparing the Performance of Data Divided Equally and Data Divided by their Similarity

- *Threaded Resnik*. We compared dividing data equally and dividing data by their similarity, using each approach with our distributed system based on (2). The average time obtained with Threaded Resnik and four slaves, and dividing data by their similarity, was reduced by an average of 92.04% (Table 19) compared with the percentage obtained by dividing data equally (Table 8). Dividing data based on their similarity reduced the average time of calculating similarity when the number of splits was increased. In other words, defining more splits resulted in splits with high similarity. This reduced the time required to calculate semantic similarity because each slave calculated similarity for a group of nodes located near each other. Using a smaller number of splits, each split still contained many unrelated or dissimilar genes; this did not reduce the average time, as was the case with more splits. Conversely, total time was increased by increasing the number of splits and slaves because of overhead during the division of GO and clustering input data (Table 20). Increasing the number of splits and slaves resulted in the highest total time in the majority of cases. In Resnik, dividing data based on their similarity did not reduce the average time in any of the test cases; this is because Resnik depends on the IC value in the calculation more than on the relationship and distance of the gene, as do other SSMs. Therefore, Resnik needs the node relationships only to find the pair ancestors and obtain the IC value of MICAs.
- *Threaded SSDD*. Using Threaded SSDD with our distributed system and input data divided by their similarity reduced the average time by an average of 24.1%, 39.2%, and 66.6% with 2, 3, and 4 slaves, respectively (Table 21). This reduction was increased by increasing the number of slaves and splits. Average time was increased with input sample size of 1000000 pairs and using 2 and 3 slaves; however, when 4 slaves were used, the average time was reduced by 38.32%. This is because defining more splits produces splits that contain genes with more similarity, relatedness, and close proximity to each other. This positively affects the average time of SSDD semantic similarity calculation, which depends on the distance and relationships of the gene. Total time was more increased if input data were divided based on their similarity rather than divided equally. This is because dividing data by their similarity requires more processing to split the GO and cluster the data. As shown in Table 22, total time was increased in most of the test cases when using 3 slaves and a sample size of 10 and 100, and when using 4 slaves and a sample size of 1000000.
- *Threaded SORA*. Using Threaded SORA in the distributed system, with input data divided by their similarity, reduced the total and average time in approximately all of the test cases (Tables 23 and 24). The average reduction percentages were approximately 33.0%, 78.2%, and 93.1% using 2, 3, and 4 slaves, respectively. An exception was observed in two cases. In the first case, there was a slight increase in average time when using two slaves and sample size of 1000. In the second case, there was a minor increase in total time when using three slaves and a sample size of 1000. As we mentioned previously, the reduction in average and total time occurs because SORA depends on calculating the distance and relationship between genes. This is affected by grouping similar and more related genes in one cluster, reducing the total and average time of calculating similarity via SORA SSM.

We compared the two methods of data allocation in the distributed system in order to measure the performance of Threaded Resnik, Threaded SSDD, and Threaded SORA. Dividing input data based on their similarity and using the data clustering algorithm gave better performance than dividing data equally. The reduction in average time more effectively reflects the performance of enhanced SSMs than does the reduction in total time. Average time reflects the time required to measure semantic similarity for the majority of gene pairs, which is not the case with total time. Total time can be increased by values that are far from the average value when calculating semantic similarity between certain genes.

In Threaded Resnik, the average time was reduced with increasing the number of slaves/splits; average time was reduced by 92.04% in the case of 4 slaves. This indicates that defining splits with more similar and related genes produces high similarity within each split and causes minimum overlap with other splits. Average time was not reduced as much as it was using other SSM. This is because, in Threaded Resnik, calculating the semantic similarity of a term depends on the number of genes annotated with it (IC value) and does not depend on its location in the GO hierarchy. In Threaded Resnik, calculating the semantic similarity of a term needs term location in the GO hierarchy only to obtain the IC value of MICA. Threaded SSDD and SORA, however, depends on the term location in the GO hierarchy. Therefore, the average time is reduced dramatically by 24.1%, 39.2%, and 66.6% in Threaded SSDD, and by 33.0%, 78.2%, and 93.1% in Threaded SORA, using 2, 3, and 4 slaves, respectively. The reduction is increased gradually by increasing the number of slaves/splits.

Total time was increased using Threaded SSDD, and markedly increased using Threaded Resnik, with increasing the number of slaves/splits. This is because the time required to run the data clustering algorithm was longer compared to that required for the semantic similarity calculation. In Threaded SORA, the time required to perform the semantic similarity calculation was very long compared to the time required to run the data clustering algorithm; therefore, total time was reduced considerably.

## 5. Conclusion

Here, we proposed a method to enhance the three best SSMs in the field of biology using parallel and distributed processing. Our approach showed a dramatic reduction in average processing time. The reduction was increased gradually by increasing the number of slaves/splits.

In Threaded Resnik, if the number of splits is small, the resulting splits contain numerous unrelated or dissimilar genes. This does not decrease the average time, as is the case with more splits. Dividing the data based on their similarity in Resnik did not reduce the average time for any of the test cases. This is because the Resnik semantic similarity measure depends on the IC value in the calculation more than on the relationship and distance of the gene. Resnik depends on term location in the GO hierarchy only to obtain the IC value of MICA. Conversely, Threaded SSDD and SORA depend on the term location in the GO hierarchy. Therefore, the average time is reduced dramatically, and the reduction is increased gradually by increasing the number of slaves/splits.

Total time was increased in Threaded Resnik and SSDD with increasing the number of slaves/splits. This is because the time required to run the GO split and data clustering algorithms is longer than that required to calculate semantic similarity. Therefore, the total time was increased considerably by increasing the number of slaves/splits. The percentage of increase in Resnik was large because the time required for semantic similarity calculation in Resnik is much less than that required by SSDD. In Threaded SORA, total time was reduced significantly. This is because, in SORA, the time required for semantic similarity calculation is very long compared to that required to run the GO split and data clustering algorithms.

These results were mainly limited by the system used to run our assessment. Our system considerably limited our ability to have more VM, processors, and RAM for each virtual machine. Provided a more powerful machine, we can complete assessments using large sample sizes, which we could not achieve in this study. So, further experiments need to be done to find the minimum and the maximum number of VMs that need to be used to enhance the performance.

In future studies, we will build a framework that will depend on the GO split and data clustering algorithms to automatically integrate big data in the field of biology. We will use Threaded Resnik, SSDD, and SORA to measure the similarity between genes and gene products, handling big data scalability and computational problems with good performance. Also, we will propose an algorithm to calculate the minimum and the maximum number of VMs that need to be used to enhance the performance.

## Figures and Tables

**Table 1:** Average time and IPs obtained using original and Threaded Resnik.

Sample Size	Original Resnik Average Time (ns)	Threaded Resnik Average Time (ns)	Improvement Percentage (IP)
10	56515	47490.44	-15.97
100	26184.95	22534.24	-13.94
1000	27907.82	21201.57	-24.03
10000	16287.99	11133.88	-31.64
100000	11844.27	7563.32	-36.14
1000000	8273.15	6179.37	-25.31
Average	24502.20	19350.47	-24.51

**Table 2:** Total time and IPs obtained using original and Threaded Resnik.

Sample Size	Original Resnik Total Time (ns)	Threaded Resnik Total Time (ns)	Improvement Percentage (IP)
10	2560906085	1776206977	-30.64
100	5350898201	6506353894	21.59
1000	5224382582	2409982084	-53.87
10000	2997898214	2691416975	-10.22
100000	9417548254	10159782237	7.88
1000000	46988654302	52629878875	12.00
Average	12090047940	12695603507	-8.88

**Table 3:** Average time and IPs obtained using original and Threaded SSDD.

Sample Size	Original SSDD Average Time (ns)	Threaded SSDD Average Time (ns)	Improvement Percentage (IP)
10	2.92E+08	8.65E+07	-70.38
100	1.32E+08	1.15E+08	-12.70
1000	9.32E+07	8.99E+07	-3.54
10000	4.62E+07	3.51E+07	-23.92
100000	4.48E+07	4.30E+07	-3.99
1000000	2.83E+07	2.18E+07	-23.01
Average	106040305	65214046.13	-22.93

**Table 4:** Total time and IPs obtained using original and Threaded SSDD.

Sample Size	Original SSDD Total Time (ns)	Threaded SSDD Total Time (ns)	Improvement Percentage (IP)
10	3124423720	1063764634	-65.95
100	14597347470	11920695608	-18.34
1000	93710848961	90410186100	-3.52
10000	4.63634E+11	3.5258E+11	-23.95
100000	4.48611E+12	4.30634E+12	-4.01
1000000	2.83292E+13	2.17894E+13	-23.09
Average	5.56506E+12	4.42528E+12	-23.14

**Table 5:** Average time and IPs obtained using original and Threaded SORA.

Sample Size	Original SORA Average Time (ns)	Threaded SORA Average Time (ns)	Improvement Percentage (IP)
10	4.14E+07	2.08E+07	-49.76
100	1.23E+08	7.71E+07	-37.39
1000	1.11E+08	7.23E+07	-34.75
10000	3.51E+09	3.06E+09	-12.81
100000	X	X	X
1000000	X	X	X
Average	9.47E+08	8.08E+08	-33.68

X indicates that, due to limited memory, the system required many hours to find the similarity of some of the pairs.

**Table 6:** Total time and IPs obtained using original and Threaded SORA.

Sample Size	Original SORA Total Time (ns)	Threaded SORA Total Time (ns)	Improvement Percentage (IP)
10	1708984548	448755395	-73.74
100	12714096406	7988678157	-37.17
1000	1.11216E+11	74128196605	-33.35
10000	3.51063E+13	3.06089E+13	-12.81
100000	X	X	X
1000000	X	X	X
Average	8.80799E+12	7.67286E+12	-39.27

X indicates that, due to limited memory, the system required many hours to find the similarity of some of the pairs.

**Table 7:** Total time obtained using a distributed system (2, 3, and 4 slaves) with Enhanced Resnik and input data divided equally.

Sample Size	Original Resnik Total Time (ns)	Threaded Resnik Total Time (ns) (Input Data Divided Equally)			% Threaded Resnik Total Time (Input Data Divided Equally) vs. Original Resnik Total Time		
		2 Slaves	3 Slaves	4 Slaves	2 Slaves	3 Slaves	4 Slaves
10	2560906085	379861713	329100621	207469547	-85.17	-87.15	-91.90
100	5350898201	234863609	223428959	403408364	-95.61	-95.82	-92.46
1000	5224382582	546062707	348324714	418142340	-89.55	-93.33	-92.00
10000	2997898214	1315383851	507249110	408547630	-56.12	-83.08	-86.37
100000	9417548254	3374684138	3254669438	3745141194	-64.17	-65.44	-60.23
1000000	46988654302	22114717802	19225589479	13719464210	-52.94	-59.08	-70.80
Average	12090047940	4660928970	3981393720	3150362214	-73.93	-80.65	-82.29

**Table 8:** Average time obtained using a distributed system (2, 3, and 4 slaves) with Enhanced Resnik and input data divided equally.

Sample Size	Original Resnik Average Time (ns)	Threaded Resnik Average Time (ns) (Input Data Divided Equally)			% Threaded Resnik Average Time (Input Data Divided Equally) vs. Original Resnik Average Time		
		2 Slaves	3 Slaves	4 Slaves	2 Slaves	3 Slaves	4 Slaves
10	56515	3.80E+08	1.48E+05	1.07E+10	672287.86	161.88	18932926.63
100	26184.94949	7.14E+04	2.90E+05	6.70E+08	172.68	1007.51	2558621.76
1000	27907.82082	3.36E+04	2.65E+04	6.46E+07	20.40	-5.04	231376.33
10000	16287.9895	1.92E+04	1.61E+04	6.45E+06	17.88	-1.15	39499.73
100000	11844.26883	7.20E+03	1.15E+04	6.56E+05	-39.21	-2.91	5438.54
1000000	8273.153824	4.31E+03	7.10E+03	7.15E+04	-47.90	-14.18	764.24
Average	24502.19708	6.34E+07	8.32E+04	1.91E+09	112068.62	191.02	3628104.54

**Table 9:** Total time obtained using a distributed system (2, 3, and 4 slaves) with Enhanced SSDD and input data divided equally.

Number of Gene Pairs	Original SSDD Total Time (ns)	Threaded SSDD Total Time (ns) (Input Data Divided Equally)			% Threaded SSDD Total Time (Input Data Divided Equally) vs. Original SSDD Total Time		
		2 Slaves	3 Slaves	4 Slaves	2 Slaves	3 Slaves	4 Slaves
10	3124423720	576766602	1100200519	668881808	-81.54	-64.79	-78.59
100	14597347470	3979998527	5141720453	2863699495	-72.73	-64.78	-80.38
1000	93710848961	28954815547	19152967373	15747791657	-69.10	-79.56	-83.20
10000	4.63634E+11	3.31738E+11	1.9269E+11	1.21739E+11	-28.45	-58.44	-73.74
100000	4.48611E+12	1.53352E+12	1.39815E+12	1.50908E+12	-65.82	-68.83	-66.36
1000000	2.83292E+13	1.65612E+13	1.25735E+13	2.0722E+13	-41.54	-55.62	-26.85
Average	5.56506E+12	3.07667E+12	2.36495E+12	3.72868E+12	-59.86	-65.34	-68.19

**Table 10:** Average time obtained using a distributed system (2, 3, and 4 slaves) with Enhanced SSDD and input data divided equally.

Number of Gene Pairs	Original SSDD Average Time (ns)	Threaded SSDD Average Time (ns) (Input Data Divided Equally)			% Threaded SSDD Average Time (Input Data Divided Equally) vs. Original SSDD Average Time		
		2 Slaves	3 Slaves	4 Slaves	2 Slaves	3 Slaves	4 Slaves
10	2.92E+08	1.55E+08	5.54E+11	1.12E+12	-46.94	189561.09	383330.36
100	1.32E+08	8.38E+07	4.01E+10	7.00E+10	-36.39	30339.33	53035.99
1000	9.32E+07	6.49E+07	3.95E+09	6.74E+09	-30.34	4139.99	7134.82
10000	4.62E+07	1.17E+08	4.38E+08	6.67E+08	153.30	848.23	1344.0
100000	4.48E+07	3.88E+07	8.03E+07	6.25E+07	-13.34	79.35	39.60
1000000	2.83E+07	4.77E+07	3.33E+07	6.68E+07	68.67	17.75	136.21
Average	1.06E+08	8.45E+07	9.98E+10	2.00E+11	1.58E+01	3.75E+04	7.42E+04

**Table 11:** Total time obtained using a distributed system (2, 3, and 4 slaves) with Enhanced SORA and input data divided equally.

Number of Gene Pairs	Original SORA Total Time (ns)	Threaded SORA Total Time (ns) (Input Data Divided Equally)			% Threaded SORA Total Time (Input Data Divided Equally) vs. Original SORA Total Time		
		2 Slaves	3 Slaves	4 Slaves	2 Slaves	3 Slaves	4 Slaves
10	1708984548	603416420	426734074	1296029007	-64.69	-75.03	-24.16
100	12714096406	4591177183	3915812000	2555994931	-63.89	-69.20	-79.90
1000	1.11216E+11	37052551034	23049069956	20918129803	-66.68	-79.28	-81.19
10000	3.51063E+13	1.23639E+13	1.2036E+13	7.32991E+12	-64.78	-65.72	-79.12
100000	X	X	X	X	X	X	X
1000000	X	X	X	X	X	X	X
Average	8.80799E+12	3.10154E+12	3.01584E+12	1.83867E+12	-65.01	-72.31	-66.09

X indicates that, due to limited memory, the system required many hours to find the similarity of some of the pairs.

**Table 12:** Enhanced SORA average time in the distributed system (2, 3 and 4 slaves) with input data divided equally.

Number of Gene Pairs	Original SORA Average Time (ns)	Threaded SORA Average Time (ns) (Input Data Divided Equally)			% Threaded SORA Average Time (Input Data Divided Equally) vs. Original SORA Average Time		
		2 Slaves	3 Slaves	4 Slaves	2 Slaves	3 Slaves	4 Slaves
10	4.14E+07	6.03E+08	5.27E+12	1.06E+13	4.82E+08	2.13E+11	1.84E+11
100	1.23E+08	7.06E+07	3.20E+11	6.63E+11	4.42E+07	1.63E+11	4.60E+10
1000	1.11E+08	6.15E+07	3.09E+10	6.40E+10	6.61E+07	6.75E+09	6.01E+09
10000	3.51E+09	1.83E+09	6.37E+09	3.56E+09	3.31E+08	6.69E+08	3.46E+08
100000	X	X	X	X	X	X	X
1000000	X	X	X	X	X	X	X
Average	9.47E+08	6.41E+08	1.41E+12	2.83E+12	2.31E+08	9.59E+10	5.91E+10

X indicates that, due to limited memory, the system required many hours to find the similarity of some of the pairs.

**Table 13:** Average time obtained using a distributed system (2, 3, and 4 slaves) with Enhanced Resnik and input data divided by their similarity.

Number of Gene Pairs	Original Resnik Average Time (ns)	Threaded Resnik Average Time (ns) (Input Data Divided by Their Similarity)			% Threaded Resnik Average Time (Input Data Divided by Their Similarity) vs. Original Resnik Average Time		
		2 Slaves	3 Slaves	4 Slaves	2 Slaves	3 Slaves	4 Slaves
10	56515	1.58E+05	3.65E+08	4.59E+08	179.57	645746.24	812073.76
100	26184.94949	6.90E+04	3.48E+07	3.52E+07	163.51	132800.77	134328.37
1000	27907.82082	3.70E+04	3.39E+06	3.41E+06	32.58	12047.13	12118.80
10000	16287.9895	2.03E+05	3.38E+05	3.47E+05	1146.32	1975.15	2030.40
100000	11844.26883	2.58E+04	3.26E+04	1.01E+04	117.83	175.24	-14.73
1000000	8273.153824	2.23E+04	2.36E+04	1.86E+04	169.55	185.26	124.82
Average	24502.19708	85850	67264033.33	82997616.67	301.56	132154.96	160110.24

**Table 14:** Total time obtained using a distributed system (2, 3, and 4 slaves) with Enhanced Resnik and input data divided by their similarity.

Number of Gene Pairs	Original Resnik Total Time (ns)	Threaded Resnik Total Time (ns) (Input Data Divided by Their Similarity)			% Threaded Resnik Total Time (Input Data Divided by Their Similarity) vs. Original Resnik Total Time		
		2 Slaves	3 Slaves	4 Slaves	2 Slaves	3 Slaves	4 Slaves
10	2560906085	335066219	211190589	3208745299	-86.92	-91.75	25.30
100	5350898201	854315104	794283629	711803925	-84.03	-85.16	-86.70
1000	5224382582	8390205103	7467893056	8870113085	60.60	42.94	69.78
10000	2997898214	93562482698	80426435081	68009514911	3020.94	2582.76	2168.57
100000	9417548254	5.81006E+11	6.31117E+11	6.47021E+11	6069.40	6601.50	6770.38
1000000	46988654302	6.55475E+12	6.35691E+12	6.12695E+12	13849.64	13428.62	12939.22
Average	12090047940	1.20648E+12	1.17949E+12	1.14246E+12	3804.94	3746.49	3647.76

**Table 15:** Average time obtained using a distributed system (2, 3, and 4 slaves) with Enhanced SSDD and input data divided by their similarity.

Number of Gene Pairs	Original SSDD Average Time (ns)	Threaded SSDD Average Time (ns) (Input Data Divided by Their Similarity)			% Threaded SSDD Average Time (Input Data Divided by Their Similarity) vs. Original SSDD Average Time		
		2 Slaves	3 Slaves	4 Slaves	2 Slaves	3 Slaves	4 Slaves
10	2.92E+08	1.24E+08	8.63E+10	1.17E+11	-57.55	29444.68	39954.78
100	1.32E+08	5.72E+07	1.04E+10	1.06E+10	-56.58	7794.49	7946.31
1000	9.32E+07	4.55E+07	1.01E+09	1.08E+09	-51.16	984.15	1059.29
10000	4.62E+07	4.47E+07	9.67E+07	1.82E+08	-3.23	109.35	294.01
100000	4.48E+07	2.47E+07	6.66E+07	4.38E+07	-44.83	48.75	-2.17
1000000	2.83E+07	6.45E+07	6.42E+07	4.12E+07	128.07	127.01	45.68
Average	1.06E+08	6.01E+07	1.63E+10	2.15E+10	-1.42E+01	6.42E+03	8.22E+03

**Table 16:** Total time obtained using a distributed system (2, 3, and 4 slaves) with Enhanced SSDD and input data divided by their similarity.

Number of Gene Pairs	Original SSDD Total Time (ns)	Threaded SSDD Total Time (ns) (Input Data Divided by Their Similarity)			% Threaded SSDD Total Time (Input Data Divided by Their Similarity) vs. Original SSDD Total Time		
		2 Slaves	3 Slaves	4 Slaves	2 Slaves	3 Slaves	4 Slaves
10	3124423720	794430504	295236711	2460790326	-74.57	-90.55	-21.24
100	14597347470	5126679035	2413422396	3134281448	-64.88	-83.47	-78.53
1000	93710848961	39111930797	36944258807	29763348812	-58.26	-60.58	-68.24
10000	4.63634E+11	4.13966E+11	3.05233E+11	3.13192E+11	-10.71	-34.17	-32.45
100000	4.48611E+12	2.48536E+12	1.92535E+12	2.06661E+12	-44.60	-57.08	-53.93
1000000	2.83292E+13	2.16457E+13	1.99122E+13	1.84843E+13	-23.59	-29.71	-34.75
Average	5.56506E+12	4.09835E+12	3.69707E+12	3.48325E+12	-46.10	-59.26	-48.19

**Table 17:** Total time obtained using a distributed system (2, 3, and 4 slaves) with Enhanced SORA and input data divided by their similarity.

Number of Gene Pairs	Original SORA Total Time (ns)	Threaded SORA Total Time (ns) (Input Data Divided by Their Similarity)			% Threaded SORA Total Time (Input Data Divided by Their Similarity) vs. Original SORA Total Time		
		2 Slaves	3 Slaves	4 Slaves	2 Slaves	3 Slaves	4 Slaves
10	1708984548	482298616	359402373	399416852	-71.78	-78.97	-76.63
100	12714096406	2840539191	2788018116	2459561951	-77.66	-78.07	-80.65
1000	1.11216E+11	28551368161	23437947918	18670965654	-74.33	-78.93	-83.21
10000	3.51063E+13	1.22567E+12	1.03491E+12	1.24612E+12	-96.51	-97.05	-96.45
100000	X	X	X	X	X	X	X
1000000	X	X	X	X	X	X	X
Average	8.80799E+12	3.14385E+11	2.65373E+11	3.16912E+11	-80.07	-83.25	-84.24

X indicates that, due to limited memory, the system required many hours to find the similarity of some of the pairs.

**Table 18:** Average time obtained using a distributed system (2, 3, and 4 slaves) with Enhanced SORA and input data divided by their similarity.

Number of Gene Pairs	Original SORA Average Time (ns)	Threaded SORA Average Time (ns) (Input Data Divided by Their Similarity)			% Threaded SORA Average Time (Input Data Divided by Their Similarity) vs. Original SORA Total Time		
		2 Slaves	3 Slaves	4 Slaves	2 Slaves	3 Slaves	4 Slaves
10	4.14E+07	4.82E+08	2.13E+11	1.84E+11	1065.48	514936.11	444813.82
100	1.23E+08	4.42E+07	1.63E+11	4.60E+10	-64.12	132207.19	37238.23
1000	1.11E+08	6.61E+07	6.75E+09	6.01E+09	-40.35	5991.10	5323.33
10000	3.51E+09	3.31E+08	6.69E+08	3.46E+08	-90.57	-80.94	-90.14
100000	X	X	X	X	X	X	X
1000000	X	X	X	X	X	X	X
Average	9.47E+08	2.31E+08	9.59E+10	5.91E+10	1065.48	1.63E+05	1.22E+05

X indicates that, due to limited memory, the system required many hours to find the similarity of some of the pairs.

**Table 19:** Average time reduction obtained using Threaded Resnik with a distributed system and input data divided by their similarity versus input data divided equally.

Number of Gene Pairs	Improvement Percentage (IP)		
	2 Slaves	3 Slaves	4 Slaves
10	-99.96	4.05	-95.71
100	-3.36	-52.76	-94.75
1000	10.12	200	-94.72
10000	957.29	1999.38	-94.62
100000	258.33	183.48	-98.46
1000000	417.40	232.39	-73.99
Average	256.64	45588.22	-92.04

**Table 20:** Total time reduction obtained using Threaded Resnik with a distributed system and input data divided by their similarity versus input data divided equally.

Number of Gene Pairs	Improvement Percentage (IP)		
	2 Slaves	3 Slaves	4 Slaves
10	-11.79	-28.59	1446.61
100	263.75	342.67	76.45
1000	1436.49	2081.14	2021.31
10000	7012.94	15755.41	16546.66
100000	17116.60	19291.13	17176.28
1000000	29539.75	32964.86	44558.83
Average	9226.29	11712.50	13637.69

**Table 21:** Average time reduction obtained using Threaded SSDD in a distributed system with input data divided by their similarity versus input data divided equally.

Number of Gene Pairs	Improvement Percentage (IP)		
	2 Slaves	3 Slaves	4 Slaves
10	-20	-84.42	-89.55
100	-31.74	-74.06	-84.86
1000	-29.89	-74.43	-83.98
10000	-61.79	-77.92	-72.71
100000	-36.34	-17.06	-29.92
1000000	35.22	92.79	-38.32
Average	-2.41E+01	-3.92E+01	-6.66E+01

**Table 22:** Total time reduction obtained using Threaded SSDD in a distributed system with input data divided by their similarity versus input data divided equally.

Number of Gene Pairs	Improvement Percentage (IP)		
	2 Slaves	3 Slaves	4 Slaves
10	37.74	-73.17	267.90
100	28.81	-53.06	9.45
1000	35.08	92.89	89.00
10000	24.79	58.41	157.26
100000	62.07	37.71	36.95
1000000	30.70	58.37	-10.80
Average	36.53	20.20	91.63

**Table 23:** Average time reduction obtained using Threaded SORA in a distributed system with input data divided by their similarity versus input data divided equally.

Number of Gene Pairs	Improvement Percentage (IP)		
	2 Slaves	3 Slaves	4 Slaves
10	-20.07	-95.96	-98.26
100	-37.39	-49.06	-93.06
1000	7.48	-78.16	-90.61
10000	-81.91	-89.50	-90.28
100000	X	X	X
1000000	X	X	X
Average	-3.30E+01	-7.82E+01	-9.31E+01

X indicates that, due to limited memory, the system required many hours to find the similarity of some of the pairs.

**Table 24:** Total time reduction obtained using Threaded SORA in a distributed system with input data divided by their similarity versus input data divided equally.

Number of Gene Pairs	Improvement Percentage (IP)		
	2 Slaves	3 Slaves	4 Slaves
10	-20.07	-15.78	-69.18
100	-38.13	-28.80	-3.77
1000	-22.94	1.69	-10.74
10000	-90.087	-91.41	-83.00
100000	X	X	X
1000000	X	X	X
Average	-42.80820245	-33.57	-41.67

X indicates that, due to limited memory, the system required many hours to find the similarity of some of the pairs.

## Data Availability

The data used to support the findings of this study are available online at [47].
